# Predictors of COVID-19-Related Perceived Improvements in Dietary Health: Results from a US Cross-Sectional Study

**DOI:** 10.3390/nu13062097

**Published:** 2021-06-19

**Authors:** Kelly Cosgrove, Christopher Wharton

**Affiliations:** Radical Simplicity Lab, College of Health Solutions, Arizona State University, 550 N 3rd Street, Phoenix, AZ 85004, USA; Christopher.Wharton@asu.edu

**Keywords:** COVID-19, nutrition, lifestyle, diet, health, United States

## Abstract

The COVID-19 pandemic resulted in substantial lifestyle changes. No US study has identified predictors of perceived dietary healthfulness changes during the pandemic period. This study included analyses of lifestyle and dietary healthfulness changes using 958 survey responses from US primary household food purchasers. Information was collected related to demographics, COVID-19-related household changes, and health-related habits before and during the pandemic. Binary logistic regression identified predictors of perceived increase in dietary healthfulness during the pandemic period. Overall, 59.8%, 16.4%, and 23.4% of participants reported that their eating habits likely changed, may have changed, and likely did not change, respectively. Of the participants whose dietary habits likely or may have changed, 64.1%, 16.8%, and 19% reported healthier, neither healthier nor less healthy, and less healthy eating habits, respectively. COVID-19-related income loss, more meals consumed with household members in front of the television, an increase in food advertisement exposure, increased perceived stress, and better perceived current health were significant predictors of a perceived increase in dietary healthfulness. Overall, dietary habits were perceived to become healthier during the pandemic. The predictors of perceived improvement in dietary healthfulness were surprising and indicate the need for further study of these factors in crisis and noncrisis situations.

## 1. Introduction

The first cases of coronavirus disease 2019 (COVID-19) were reported in Hubei Province in the People’s Republic of China and were soon reported in many other countries, which caused the World Health Organization (WHO) to declare a state of emergency on 30 January 2020 [1]. After the US exhibited a marked increase in COVID-19 cases, a nationwide “stay-at-home” order was issued on 16 March 2020; public schools were closed, and restrictions were placed on various businesses and public gatherings [2]. Those and other efforts to contain the spread of COVID-19 likely resulted in substantial lifestyle changes in households in the US and worldwide. 

Many previously reported COVID-19-related lifestyle changes, such as transitioning to telecommuting, preparing more meals at home, and modifying food procurement habits, have likely led to shifts in dietary habits during the COVID-19 pandemic period [3,4,5]. Shifts in dietary habits are of great importance in the US, where the majority of deaths and health care expenditures are attributed to diet- and lifestyle-related chronic diseases [6]. Moreover, diet has become increasingly important during the COVID-19 pandemic because of the impacts of nutrition on chronic conditions known to lead to worse COVID-19 outcomes as well as on immune function, which could impact the ability of the body to resist infection with severe acute respiratory syndrome coronavirus 2 (SARS-CoV-2), the virus that causes COVID-19 [7,8]. Additionally, trans-membrane angiotensin-converting enzyme 2 (ACE2) facilitates the entry of SARS-CoV-2 into cells, and various dietary compounds and patterns have been found to be associated with ACE levels [9]. For example, quercetin and other polyphenols, which are found mostly in plant-based foods such as berries, herbs, beans, and nuts, have been found to inhibit ACE2 activity [10]. 

A number of COVID-19-related factors identified in previous research could be expected to impact dietary intake [11,12,13]. For example, individuals worldwide have been experiencing high levels of stress due to factors associated with the pandemic, such as fear of contracting COVID-19, employment loss, and lack of socialization [14]. Psychological stress has been associated with greater consumption of food, particularly high-fat foods [15]. It has also been posited that boredom caused by remaining at home during the pandemic could lead to worse diet quality and promote overconsumption [12].

COVID-19-related changes in food procurement could also be expected to impact dietary habits during the pandemic. For many in the US in particular, access to restaurants was restricted or eliminated, and many people have changed their grocery shopping habits; for example, some households have begun purchasing groceries online to avoid coming into contact with people in the grocery store and some households have started purchasing more food during each shopping trip to limit the number of trips to the store [16]. These changes in shopping habits could have important impacts on diet.

Income loss associated with the pandemic has also put a strain on many individuals worldwide, and financial limitations are important to consider when assessing dietary changes related to the pandemic. In the US, food insecurity increased markedly during the COVID-19 pandemic. Many households experiencing food insecurity reported difficulties complying with the recommendations to purchase two weeks of food at a time to limit trips to the grocery store [17]. Food insecurity has been found to be inversely associated with diet quality, which could be explained by the fact that healthier foods tend to be more expensive or that experiencing food insecurity leads to psychological stress, which may cause individuals to consume highly-palatable foods that are higher in fat and sodium [18]. 

Since the early stages of the pandemic, there has been an emphasis on the importance of conducting observational research to examine the impacts of the COVID-19 pandemic on lifestyle behaviors [19]. Accordingly, studies examining various COVID-19-related lifestyle changes have been conducted in several countries, such as Italy, China, India, and Spain [20,21,22,23]. Additionally, lifestyle changes have been explored in specific populations, such as college students, individuals with diabetes, pregnant women, and children [24,25,26,27]. Moreover, the majority of studies assessing COVID-19-related changes in lifestyle behaviors have largely focused on physical activity and sedentary behavior, neglecting the important influence of diet during this health crisis. To the authors’ knowledge, no study conducted in the US to date has examined COVID-19-related perceived changes in dietary healthfulness in the general US population. Therefore, this study aimed to explore this topic and identified multiple predictors of perceived improvement in dietary healthfulness among US households. 

## 2. Materials and Methods

### 2.1. COVID-19 and Health Behaviors Questionnaire

A 57-item questionnaire was used to collect information on individual demographic factors of the household food purchaser (e.g., age, sex, education), household characteristics (e.g., income, household size, number of children under the age of 18 years), COVID-19-related household changes (e.g., children staying at home because of the COVID-19 pandemic, changes in employment income as a result of COVID-19), changes in food-related behaviors due to the pandemic (e.g., food procurement locations, frequency of home food preparation), and health-related factors (e.g., perceived stress (assessed using the widely used perceived stress scale (PSS) [28]), changes in dietary healthfulness, and screen time behaviors). The questionnaire was developed by the research team using validated or widely used questions from surveys used to collect similar information when possible. For example, demographic and household items reflected those used in the US Census Pulse Surveys to gather similar data [29]. Questions that were unique to the circumstances related to COVID-19 and that were not obtained from existing questionnaires were assessed for clarity and comprehensibility by at least three investigators with experience designing surveys.

To assess changes in perceived dietary healthfulness, participants were first asked “did the eating habits of anyone in your household change because of the COVID-19 pandemic?” The response options were as follows: “definitely yes,” “probably yes,” “might or might not,” “probably not,” and “definitely not.” Individuals who did not select either “definitely not” or “probably not” were then asked “how would you describe the impact of those changes on the healthfulness of eating habits in your household?” The participants could then respond that the eating habits in their household became much healthier, somewhat healthier, neither healthier nor less healthy, somewhat less healthy, or much less healthy. The survey is available in the Supplementary Materials (File S1). 

### 2.2. Data Collection

The data collection methods have been described in detail previously [30]. Briefly, participants recruited via Amazon Mechanical Turk (MTurk) completed a survey that was delivered online through Qualtrics. Previous studies have indicated that individuals residing in the U.S. who complete surveys on MTurk are more similar to the U.S. internet population and more diverse than traditional pools of subjects (e.g., college students) [31]. 

Survey responses were collected on 2 October 2020, approximately seven months after US lockdowns began, allowing for the assessment of longer-term lifestyle changes associated with the pandemic. This study was approved by the Institutional Review Board of Arizona State University (protocol code STUDY00012660), and all participants provided informed consent prior to the completion of the survey.

### 2.3. Statistical Analyses

Categorical data are presented as frequencies and percentages. A binary logistic regression analysis was conducted to examine which household factors predicted a perceived increase in dietary healthfulness. The variables included in the model were as follows: change in the frequency of consuming meals in front of the television, perceived stress, household food purchaser sex, 2019 household income, COVID-19-related income loss, change in exposure to food advertisements, shift to telecommuting during the pandemic, and perceived current health. Multicollinearity among these variables was assessed using variance inflation factors (VIFs), and a linear relationship between the continuous predictor (PSS score) and its log odds was confirmed. All statistical analyses were conducted using SPSS v. 27 (IBM Corp., Armonk, NY, USA). Statistical significance was set at alpha level <0.05. 

## 3. Results

### 3.1. Demographics and Household Characteristics

In total, 958 survey responses were obtained from self-identified household food purchasers residing in the US. Within the sample, the majority of respondents were male (51.3%), had a bachelor’s degree (58.9%), and were married (65.4%) (Table 1). A preponderance of households reported incomes between $50,000 and $74,999 (28.8%), four household members (29.0%), and zero children (38.2%) (Table 2). The sex and income distributions of the study sample were similar to those of the general US population according to 2019 US Census data. However, the study sample had a higher education level and a higher prevalence of married individuals.

### 3.2. COVID-19-Related Household Changes

**Figure 1:**
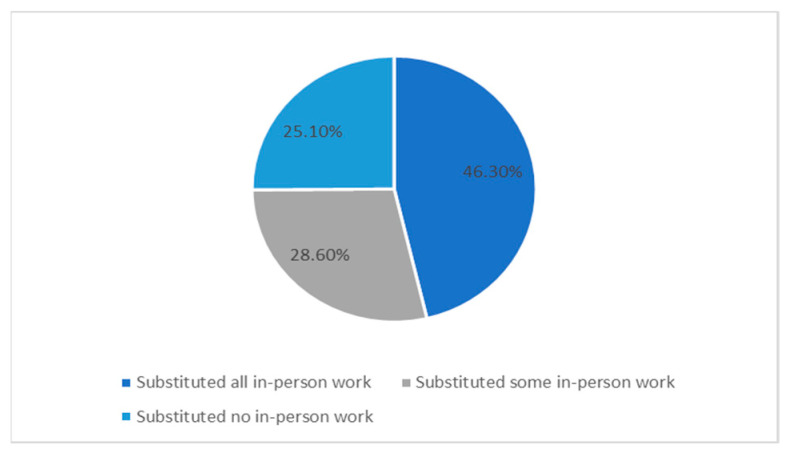
Transition to telecommuting among the participating households.

Participants were asked about ways in which their household was impacted by the COVID-19 pandemic. Overall, 481 (50.2%) households reported that someone in their household experienced income loss associated with the pandemic. In 444 (46.3%) households, at least one adult substituted all of their in-person work for telework (working from home); in 274 (28.6%) households, at least one adult substituted some of their in-person work for telework; and in 240 (25.1%) households, no adults substituted in-person work for telework (Figure 1). Additionally, in 86.7% of households with children, an adult was responsible for caring for children who were staying home because of the COVID-19 pandemic. 

### 3.3. COVID-19-Related Health Situation

**Figure 2:**
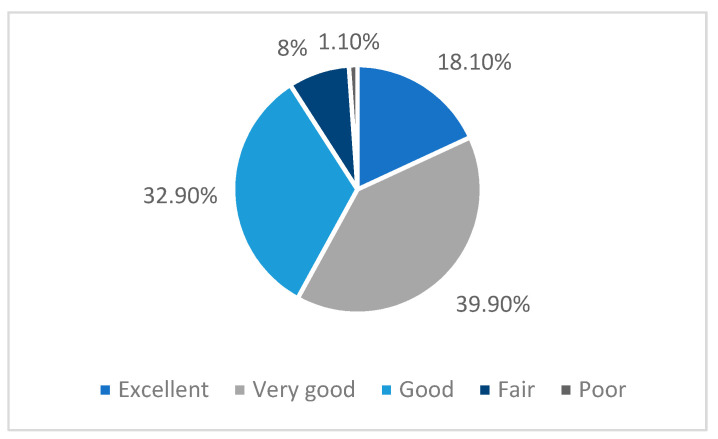
Perceived health during the COVID-19 pandemic.

Overall, during the pandemic period, the survey respondents reported good perceived health: 173 (18.1%) reported excellent health, 382 (39.9%) reported very good health, 315 (32.9%) reported good health, 77 (8.0%) reported fair health, and 11 (1.1%) reported poor health (Figure 2). 

The survey respondents also completed the perceived stress scale (PSS), which revealed that 225 (23.5%) participants were experiencing low stress (PSS scores ranging from 0 to 13), 681 (71.1%) were experiencing moderate stress (PSS scores ranging from 14 to 26), and 52 (5.4%) were experiencing high stress (PSS scores ranging from 27 to 40).

**Figure 3:**
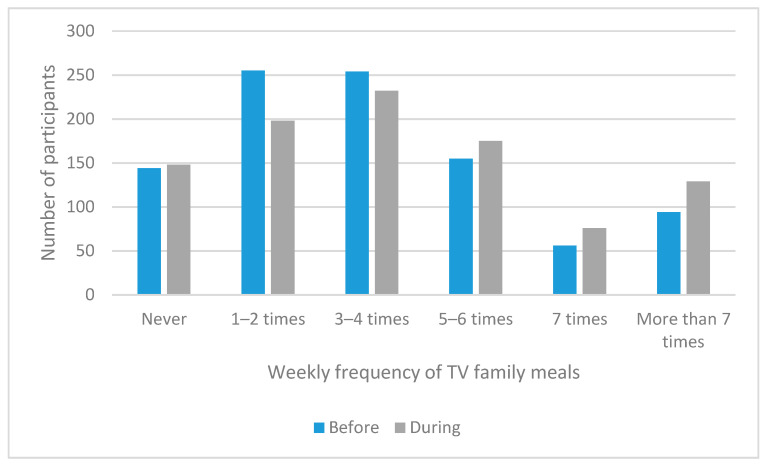
Weekly frequency of consuming meals with all or most of the people in the household while watching television prior to and during the pandemic.

Prior to the pandemic, 144 (15.0%), 255 (26.6%), 254 (26.5%), 155 (16.2%), 56 (5.8%), and 94 (9.8%) of participants reported that in a typical week, all or most of the people in the household consumed a meal together while watching television never, 1–2 times, 3–4 times, 5–6 times, 7 times, and more than 7 times, respectively, compared to 148 (15.4%), 198 (20.7%), 232 (24.2%), 175 (18.3%), 76 (7.9%), and 129 (13.5%), respectively, during the pandemic (Figure 3). Regarding the change in the frequency of consuming meals with household members in front of the television, 163 (17.0%) reported a decrease in frequency, 521 (54.4%) reported no change in frequency, and 274 (28.6%) reported an increase in frequency. 

### 3.4. COVID-19-Related Food Behavior Changes

#### 3.4.1. Food Security

**Figure 4:**
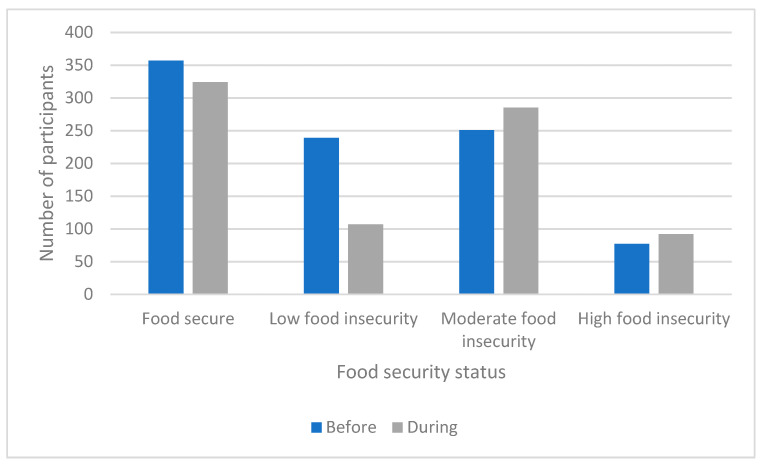
Food security status among the households prior to and during the pandemic.

Prior to the pandemic (*n* = 924), 357 (38.6%) participating households perceived they were food secure, and 239 (25.8%), 251 (27.2%), and 77 (8.3%) participating households reported experiencing low, moderate, and high levels of food insecurity, respectively. During the pandemic period (*n* = 925), 324 (35.0%) households identified as food secure, and 107 (24.3%), 285 (30.8%), and 92 (9.9%) participating households reported experiencing low, moderate, and high levels of food insecurity, respectively (Figure 4).

#### 3.4.2. Food Procurement 

**Figure 5:**
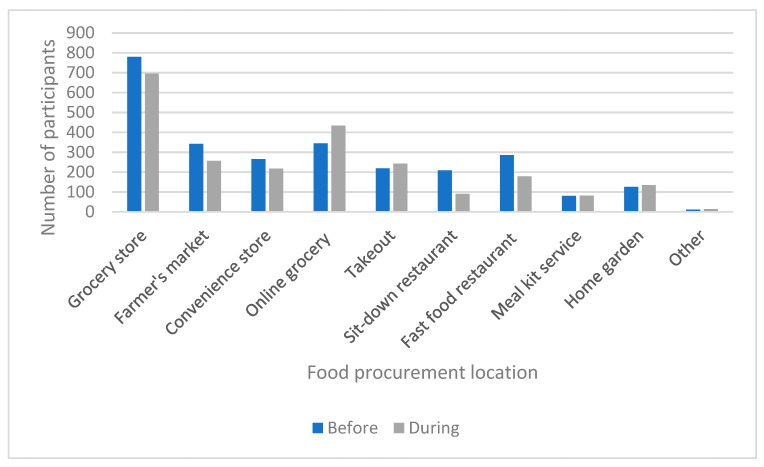
Most common food procurement locations prior to and during the pandemic.

Participants were asked to report where they obtained most of the food for household consumption both prior to and during the COVID-19 pandemic period. Participants selected one or more of the following options: grocery store, farmer’s market, convenience store, online grocery, takeout, sit-down restaurant, fast food restaurant, meal kit service, home garden, and other (Figure 5). Notable changes occurred in relation to obtaining food from grocery stores, farmer’s markets, sit-down restaurants, and fast food restaurants, all of which seemed to decrease. Increases were observed in online grocery shopping and ordering takeout. 

#### 3.4.3. Changes in Grocery Shopping Habits by Food Category

**Figure 6:**
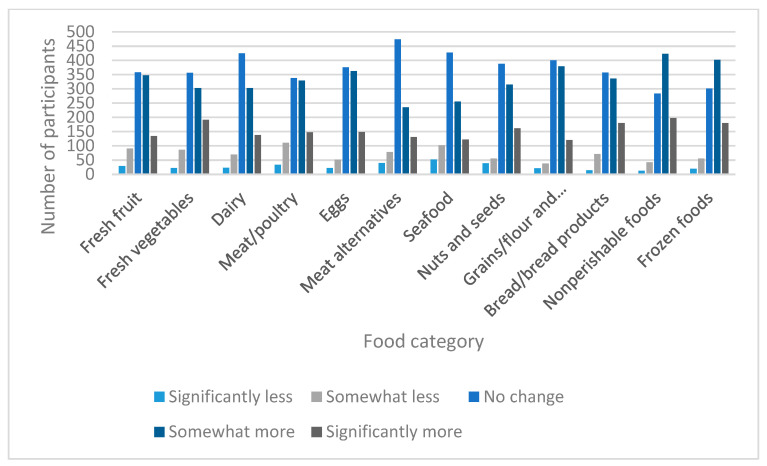
Changes in grocery shopping behaviors during the COVID-19 pandemic.

Participants were asked to estimate how their shopping habits changed for various food categories during the COVID-19 pandemic relative to their habits before the pandemic. The response options for the different food groups were as follows: “Buy significantly less,” “Buy somewhat less,” “No change,” “Buy somewhat more,” and “Buy significantly more.” Overall, participants most often reported purchasing more food in each food category, with only slight differences in response frequencies among food categories (Figure 6).

#### 3.4.4. Fast Food Consumption 

**Figure 7:**
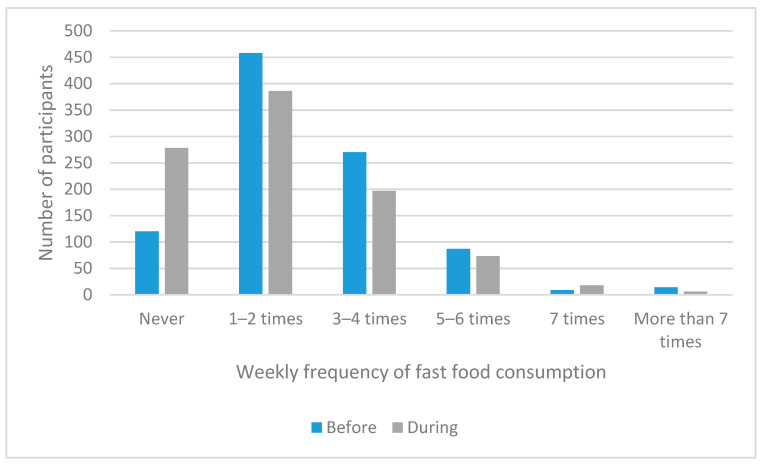
Weekly fast food consumption frequency prior to and during the pandemic.

The frequency of fast food consumption was explored in greater depth. Participants were asked to report the average weekly frequency of fast food consumption prior to and during the pandemic period. The results indicated an overall reduction in the consumption of fast food (Figure 7), which is consistent with the abovementioned results of the food procurement analysis. 

#### 3.4.5. Food Advertisements

**Figure 8:**
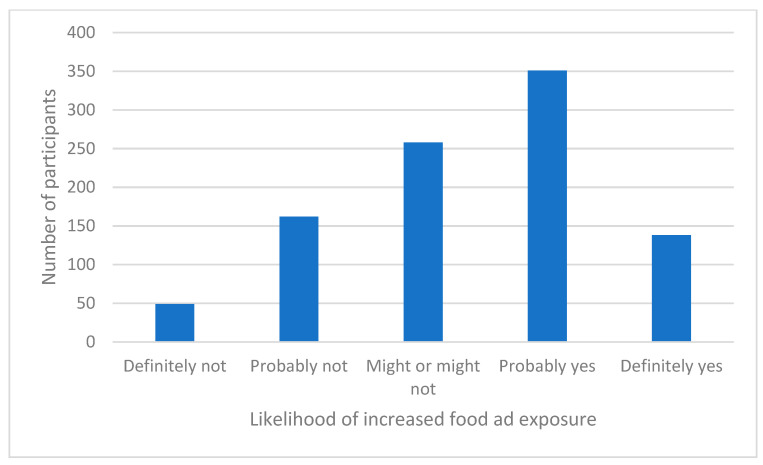
Perceived likelihood of increased exposure to food advertisements among household members.

The perceived likelihood of increased exposure to food advertisements among household members was assessed. As shown in Figure 8, overall, respondents reported that the individuals in their household were likely exposed to more food advertisements during the COVID-19 pandemic period. 

#### 3.4.6. Change in Dietary Habits

**Figure 9:**
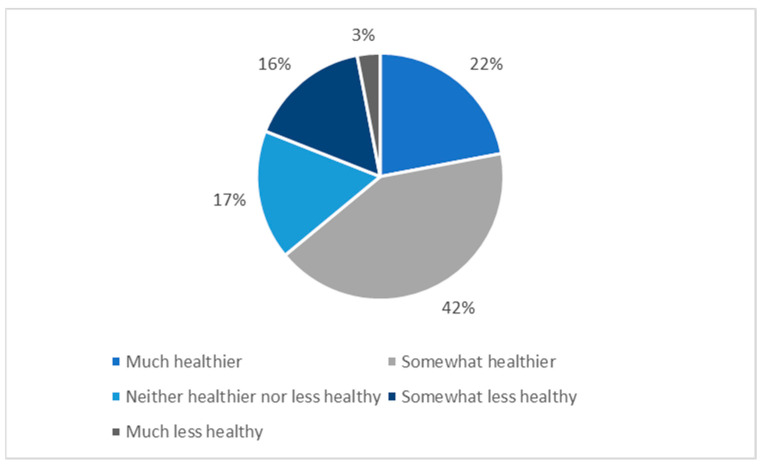
Perceived impact of COVID-19-related dietary changes on dietary healthfulness.

Participants were asked whether the eating habits of anyone in their household changed as a result of the COVID-19 pandemic. A total of 181 (18.9%) answered ‘definitely yes,’ 392 (40.9%) answered ‘probably yes,’ 157 (16.4%) answered ‘might or might not,’ 138 (14.4%) reported ‘probably not,’ and 90 (9.4%) reported ‘definitely not.’ Participants who answered ‘might or might not,’ probably yes,’ or ‘definitely yes,’ were asked to describe the impact of the potential dietary changes on the healthfulness of eating habits in their household. A total of 158 (16.5%) participants said that eating habits became much healthier, 310 (32.4%) said they became somewhat healthier, 123 (12.8%) said they became neither healthier nor less healthy, 120 (12.5%) said they became somewhat less healthy, and 19 (2.0%) said they became much less healthy (Figure 9).

**Figure 10:**
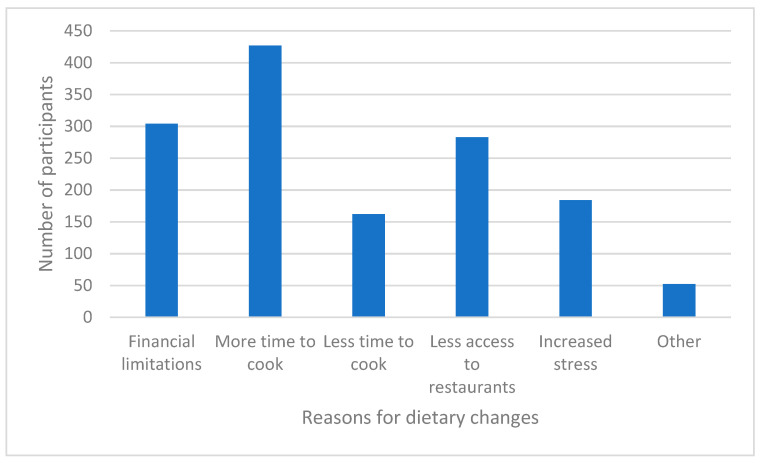
Reasons for perceived COVID-19-related dietary changes in the household.

Participants were asked to select all of the likely reasons for the changes in dietary habits in their household from the following options: financial limitations, more time to cook, less time to cook, less access to restaurants, increased stress/emotional eating, and other (open response). The results are shown in Figure 10. The most influential factors were found to be more time to cook, financial limitations, and less access to restaurants. Participants who selected ‘other’ were able to provide more information in the form of a free response. Several responses indicated that participants desired to improve their health to avoid contracting COVID-19 or to use their extra time to work towards their long-standing health goals. Other responses centered around the theme of boredom. Participants reported eating more because they felt bored. There were also responses related to increased financial capabilities due to the enhanced unemployment benefits as well as increased food consumption resulting from working at home. 

### 3.5. Predictors of a Perceived Increase in Dietary Healthfulness

Binary logistic regression was conducted to identify predictors of a perceived increase in dietary healthfulness. Perceived dietary healthfulness was coded into a binary variable: all participants who responded ‘somewhat healthier’ or ‘much healthier’ to the question ‘How would you describe the impact of those changes on the healthfulness of eating habits in your household?’ were given a value of ‘1,’ and all other participants were assigned a value of ‘0.’ There was no evidence of multicollinearity among the predictor variables according to VIFs (all < 1.2). The model was significant (χ^2^(8) = 153.138, *p* < 0.001). The model explained 19.7% of the variance in perceived increase in dietary healthfulness. The following variables were found to significantly predict increased odds of a perceived increase in dietary healthfulness: increase in frequency of consuming meals with family in front of the TV (OR = 1.18, 95% CI 1.04–1.34, *p* = 0.009), COVID-19-related income loss (OR = 1.64, 95% CI 1.22–2.21, *p* < 0.001), increased exposure to food advertisements (OR = 1.52, 95% CI 1.32–1.74, *p* < 0.001), increased PSS score (OR = 1.04, 95% CI 1.02–1.07, *p* < 0.001), and better perceived current health (OR = 1.53, 95% CI 1.29–1.81, *p* < 0.001) (Table 3).

#### 3.5.1. Subgroup Analyses According to Sex

The same regression analysis (excluding sex as an independent variable in the model) was conducted separately for males and females. The statistical conclusions of the male-only regression differed slightly from those of the regression conducted in the total sample. Change in frequency of meals consumed with the family in front of the television was not a significant predictor of perceived increase in dietary healthfulness in males (OR = 1.05, 95% CI = 0.88–1.25, *p* = 0.601), and PSS score achieved only borderline significance in the male-only regression (OR = 1.03, 95% CI = 1.00–1.07, *p* = 0.05) (Supplementary Table S1). The statistical conclusions of the female-only regression were consistent with those of the regression conducted in the total sample (Supplementary Table S2). 

#### 3.5.2. Subgroup Analyses According to Age Group

The regression analysis was then conducted separately by age group. These findings are intended for descriptive purposes only because the reduced power of the subgroup analyses limit comparability with the results of the regression conducted with the total sample. Therefore, these results should be interpreted with caution. 

The statistical conclusions of the regressions conducted separately by age group differed slightly from those of the regression conducted in the total sample. In individuals 18–29 years of age, change in food ad exposure and PSS score were not significant predictors of perceived increase in dietary healthfulness (OR = 1.15, 95% CI = 0.82–1.61, *p* = 0.424 and OR = 1.04, 95% CI = 0.98–1.11, *p* = 0.216, respectively) (Supplementary Table S3). In individuals 30–39 years of age, change in frequency of consumption of meals with family in front of the television, COVID-19-related income loss, and PSS score were not significant predictors of perceived increase in dietary healthfulness (OR = 1.09, 95% CI = 0.89–1.32, *p* = 0.402; OR = 1.27, 95% CI = 0.78–2.07, *p* = 0.332; and OR = 1.04, 95% CI = 1.00–1.08, *p* = 0.072, respectively) (Supplementary Table S4). In individuals 40–49 years of age, change in frequency of consumption of meals with family in front of the television and COVID-19-related income loss were not significant predictors of perceived increase in dietary healthfulness (OR = 1.13, 95% CI = 0.84–1.54, *p* = 0.422 and OR = 1.40, 95% CI = 0.71–2.75, *p* = 0.327, respectively) (Supplementary Table S5). In individuals aged 50–59 years, change in frequency of consumption of meals with family in front of the television, COVID-19-related income loss, PSS score, and perceived current health were not significant predictors of perceived increase in dietary healthfulness (OR = 1.26, 95% CI = 0.80–2.00, *p* = 0.313; OR = 2.33, 95% CI = 0.93–5.80, *p* = 0.070; OR = 1.06, 95% CI = 0.99–1.14, *p* = 0.083; and OR = 1.50, 95% CI = 0.87–2.57, *p* = 0.142, respectively) (Supplementary Table S6). In individuals aged 60+ years, change in frequency of consumption of meals with family in front of the television, PSS score, and perceived current health were not significant predictors of perceived increase in dietary healthfulness (OR = 0.91, 95% CI = 0.57–1.47, *p* = 0.705; OR = 1.00, 95% CI = 0.93–1.08, *p* = 0.938; OR = 0.96, 95% CI = 0.57–1.63, *p* = 0.876, respectively) (Supplementary Table S7). 

#### 3.5.3. Associations of COVID-19-Related Income Loss with Reasons for Dietary Change

Because the most powerful predictor of improved dietary healthfulness was found to be COVID-19-related income loss in the total sample, which was unexpected, the associations of COVID-19-related income loss with the reported reasons for dietary changes in the household were assessed using a chi square test. COVID-19-related income loss was found to be significantly associated with the following responses to the question ‘Why did eating habits change in your household? Check all that apply’: financial limitations (χ^2^(1) = 50.854, *p* < 0.001), more time to cook (χ^2^(1) = 32.141, *p* < 0.001), and less time to cook (χ^2^(1) = 26.146, *p* < 0.001). However, significant associations were not found for the remaining options: less access to restaurants (χ^2^(1) = 2.845, *p* = 0.092) and increased stress/emotional eating (χ^2^(1) = 0.07, *p* = 0.791).

## 4. Discussion

The COVID-19 pandemic has resulted in dramatic changes in the lives of people worldwide. At the household level, there have been mixed findings regarding the impacts of the pandemic. Some studies have suggested improvements in certain habits, such as reductions in fast food consumption and increases in home-prepared meals [32]. However, other studies revealed negative effects of the pandemic on lifestyle. For example, the dietary habits, activity levels, and screen-time habits of obese children in Italy were found to be adversely impacted by the COVID-19 lockdown [33]. An international survey study also revealed negative consequences of the COVID-19 pandemic on mental health and lifestyle behaviors [34]. Moreover, in a sample of patients with chronic coronary syndromes, widespread reductions in physical activity and increases in tobacco use and weight were reported [35]. In the current study, certain adverse effects of the pandemic, such as income loss and stress, were reported by the study participants. However, positive changes during the pandemic period were also reported. For example, the majority of participants reported improvements in dietary healthfulness during the pandemic period. This finding was different from the results of a French study that revealed that diet quality worsened during the pandemic period. Those investigators showed that food choice motives were important factors related to changes in diet quality. For example, improved diet quality was associated with an increase in the importance of weight control, and reduced diet quality was associated with increased importance of mood [36].

The current study found that the majority of participants were experiencing moderate to high stress levels during the pandemic period. This finding is consistent with previous research conducted during the COVID-19 pandemic period in other countries, such as the US [14], the Philippines [37], China [38], and Bangladesh [39]. However, despite the high levels of stress, most participants reported excellent or very good health. A study conducted in Germany approximately two months after the implementation of COVID-19-related restrictions revealed that 32% of participants reported improvements in perceived health [40]. Additionally, a study conducted in France indicated that compared to previous years, self-reported health and well-being improved during the pandemic period overall [41]. The researchers identified divergences in the effects on well-being and self-reported health related to type of employment, area of residence, and working hours. These findings, along with the findings of the current study, indicate the need for further exploration as it would be expected that the reductions in physical activity and socialization as well as the stress associated with the pandemic might lead to worse perceived health.

Even so, a number of potential explanations for the high ratings of perceived health and improvements in dietary healthfulness during the COVID-19 pandemic period are possible. The food environment is highly associated with health outcomes. The types of stores and restaurants at which individuals obtain food have been found to be an important influencer of food choices and diet-related health outcomes [42]. As evidenced by the results of the present study, food shopping habits have been widely impacted by measures to prevent the spread of COVID-19. In particular, increases in online grocery shopping could have led to changes in food choices because of a reduction in environmental influences that are more prevalent in in-person food shopping experiences. Previous research has shown that contextual factors can contribute to unhealthy food choices over healthy ones. A recent study that examined how food choices differ when individuals shop online rather than in a physical store revealed that consumers purchased fewer unhealthy products when shopping online. The investigators indicated that this difference in food choice may occur because decreases in products’ ‘vividness’ associated with their online presentation diminish consumers’ desires for instant gratification [43]. This concept deserves further attention as it has important implications for consumers, policy makers, and retailers in both crisis and noncrisis situations. 

While the majority of participants in the current study reported similar shifts in their grocery shopping habits for all food categories during the pandemic period, it is difficult to tie these shifts to the reported perceived changes in dietary healthfulness. These data were collected to identify any potential large shifts in dietary patterns (e.g., shifts to a more plant-based diet) and to inform a previously published study focused on household food waste [30]. Overall, the majority of participants reported purchasing more food in all categories assessed with similar trends found for each food category. This is likely explained by the fact that participants were purchasing more food from the grocery store overall due to the reduction in meals consumed away from the home. The food categories assessed were not specific enough to yield any conclusions on healthfulness of the foods purchased within each category. For example, with the exception of the categories of fresh fruits and fresh vegetables, which are overwhelmingly considered healthy dietary components, other food categories assessed, such as meat/poultry and grains/flour and legumes, can include both healthier and less healthy options, and participants who reported consuming healthier diets during the pandemic period could have opted for healthier options within each category. Therefore, the assessment of these food categories cannot yield valid nutritional conclusions. 

In addition to the potential impact of changes in food shopping habits on food choices, in the current study, COVID-19-related income loss, an increased number of meals consumed with household members in front of the television, increased food advertisement exposure, increased perceived stress, and better perceived current health were found to be significant predictors of a perceived increase in dietary healthfulness during the pandemic. The result indicating that COVID-19-related income loss was associated with a perceived increase in dietary healthfulness is not consistent with the findings of a study conducted among pregnant women in the US earlier in the pandemic period; the study conducted among pregnant women indicated that loss of income due to COVID-19 was associated with adverse lifestyle changes, including the adoption of worse dietary habits [44]. However, circumstances changed rapidly as the pandemic wore on, and some of those changes might have played a role in the results of this study, which was conducted later in the pandemic. For example, the federal Coronavirus Aid, Relief, and Economic Security (CARES) Act was signed into law on 27 March 2020 [45]. The Act provided a $600/week supplement to state unemployment benefits. The Act was designed to alleviate concern over the ability to pay bills and cover necessities, such as food purchases, and may have influenced how people shopped once benefits were in place [46]. 

Some have also argued that the relationship between income and diet quality is weak and that at all spending levels, there are households that consume healthy and unhealthy diets [47]. A study examining the impacts of the Great Recession on the healthfulness of foods purchased in the US revealed that the recession resulted in a 4–8% increase in diet quality according to grocery store purchases [48]. Moreover, during the Great Recession, consuming food away from home decreased in favor of consuming more home-prepared meals; this shift led to improvements in diet quality as food away from home is often associated with lower diet quality [49]. However, the improvements in diet quality observed during the Great Recession were not fully explained by the reduction in eating out, indicating that other factors such as greater focus on nutrition and improved nutritional quality of foods likely contributed to the nutritional shift. This area of research deserves greater focus as income is commonly considered an important predictor of the ability to consume a healthy diet. However, some research has provided evidence to the contrary [47,50], which could have important implications for policy and allocation of resources to encourage the adoption of healthier dietary habits among individuals with lower socioeconomic status. That research has suggested that factors associated with socioeconomic status, such as stress, schedule, and education, may be more important predictors of dietary healthfulness than income itself.

The identification of an increase in the number of meals consumed with household members in front of the television as a significant predictor of a perceived improvement in dietary healthfulness was also surprising. During the Great Recession, it was found that time available to prepare food at home and eat meals together as a family increased as a result of increased unemployment, which led to improvements in diet quality [49]. While that research did not examine the effects of television and screen time during meals, the increase in the frequency of family meals might be the greater factor at play in this study, driving increased frequency of meals consumed with household members in front of the television yet still contributing to perceived improvements in diet quality. 

Increased exposure to food advertisements was not expected to be a predictor of perceived improvements in dietary healthfulness. Possible explanations for this association include exposure to advertisements of healthier foods or reduced influence of food advertisements due to the strength of the other pandemic-related food influences. The content of food advertisements targeting adults requires further research, as studies on food advertisements have mostly focused on children [51,52,53]. Moreover, the advertising environment has changed markedly in recent years, with an increase in advertisements on social media and the use of personal data and algorithms to provide targeted advertisements. This new era of advertising has many important implications for dietary choices. 

Higher levels of perceived stress were identified as a predictor of a perceived improvement in dietary healthfulness. While increased psychological stress is commonly associated with reduced diet quality [54], there is limited research on the impact of stress resulting from a health-related crisis on diet quality. It could be posited that experiencing stress due to fear of contracting a disease could lead to healthier lifestyle choices. This area requires further research. However, the results of the present study indicated the magnitude of the relationship between perceived stress and perceived improvement in dietary healthfulness was small (OR = 1.04, 95% CI 1.02–1.07); therefore, the impact of perceived stress on perceived dietary habits was likely not substantial in the study sample. 

Overall, the increase in perceived dietary healthfulness could be explained by individuals attempting to improve their health to avoid contracting COVID-19. A study that examined popular search terms before and during the COVID-19 pandemic indicated an increase in searches including health-related terms, such as exercise, fitness, vitamins, zinc, and turmeric, after the start of the pandemic [55]. This increase in health-related searches could have also led to increased advertisements of healthier foods due to targeted advertisements based on web-browsing history. During the COVID-19 pandemic period, Polish adults who were overweight or obese prior to the pandemic were found to be more likely to improve the healthfulness of their diet during the pandemic period [12]. This finding is consistent with the qualitative data collected in the present study indicating that people were using their extra free time to improve their health and work toward their long-standing health goals. These findings have important implications because they indicate that given the time and schedule flexibility, people are likely to take initiative to improve their health behaviors. 

This study benefited from a number of strengths, including the relatively large national sample. In addition, to the authors’ knowledge, this is the first US study to examine the factors associated with improvements in perceived dietary healthfulness during the COVID-19 pandemic period. This is an important area of research because diet quality is overwhelmingly low in the US, and many of the leading causes of death and health care costs are caused by diet-related chronic diseases [56]. Therefore, improving diet quality in the US is an important focus, and identifying factors that led to perceived improvements in dietary healthfulness during the pandemic period could inform future research on encouraging the adoption of healthier dietary patterns. 

This study also suffered from some limitations. This was a cross-sectional study, which prevented the establishment of causal relationships among the factors analyzed. Moreover, all of the information collected was self-reported, which could lead to biased data. Additionally, specific dietary data were not collected in the present study, and the assessment of dietary healthfulness relied solely on the participants’ perceptions of the healthfulness of their diet compared to that before the pandemic period. Because participants were asked to compare their habits during the pandemic period to those prior to the pandemic period, recall bias could have led to inaccurate responses. The subgroup analyses conducted were underpowered, and the results were reported for descriptive purposes only. Therefore, the results of the subgroup analyses should be confirmed in additional studies powered for such subgroup analyses. 

Additionally, pandemic-related changes in programs developed to improve population health, such as access to the National School Lunch Program in the US, were not assessed in this study. Although some such programs were allowed to remain open despite the closure of schools and other institutions, any changes that might have occurred could have substantial long-term health effects, especially among lower-income individuals who rely more heavily on these programs to obtain nutritious food. Despite these limitations, this study provided valuable information related to dietary changes associated with crisis situations in the US. Future studies should examine changes in dietary healthfulness associated specifically with health-related stress as well as the impacts of income loss and financial hardship on dietary healthfulness. 

## 5. Conclusions

This study revealed that dietary habits were perceived to improve overall during the COVID-19 pandemic, indicating that people were making an effort to improve their health during this unprecedented health crisis. Because the adoption of healthy behaviors is essential for improved health and well-being and disease prevention, exploring lifestyle factors that may have led to the adoption of healthier behaviors during this period of disruption can allow for more effective crisis response in the future and could also reveal important areas for further research on behavior change and preventative health.

## Figures and Tables

**Table 1:** Demographic characteristics of US adults who completed the COVID-19 and Lifestyle Questionnaire (*n* = 958).

	Frequency	Percentage
Sex		
Male	491	51.3
Female	467	48.7
Age range (years)		
18–29	205	21.4
30–39	331	34.6
40–49	200	20.9
50–59	120	12.5
60+	102	10.6
Education		
Less than high school	3	0.3
Some high school	1	0.1
High school graduate	51	5.3
Some college	109	11.4
Associate’s degree	83	8.7
Bachelor’s degree	564	58.9
Graduate degree	147	15.3
Marital status		
Married	627	65.4
Widowed	24	2.5
Divorced	63	6.6
Separated	15	1.6
Never married	229	23.9

**Table 2:** Household characteristics of US adults who completed the COVID-19 and Lifestyle Questionnaire (*n* = 958).

	Frequency	Percentage
Household income		
Less than $25,000	125	13.0
$25,000–$34,999	91	9.5
$35,000–$49,999	173	18.1
$50,000–$74,999	276	28.8
$75,000–$99,999	156	16.3
$100,000–$149,999	97	10.1
$150,000–$199,999	23	2.4
$200,000+	17	1.8
Household size		
1	130	13.6
2	220	23.0
3	184	19.2
4	278	29.0
5+	144	15.0
Number of children		
0	366	38.2
1	264	27.6
2	248	25.9
3	41	4.3
4+	39	4.1

**Table 3:** Binary logistic regression analysis of perceived increase in dietary healthfulness (*n* = 958).

Independent Variable	B	S.E.	Wald	*p*-Value	OR (95% CI)
Sex	−0.16	0.14	1.22	0.27	0.85 (0.65–1.13)
Household income (2019)	0.02	0.04	0.28	0.597	1.02 (0.94–1.12)
Change in frequency meals with family in front of the TV	0.17	0.06	6.79	0.009	1.18 (1.04–1.34) *
COVID-19 income loss	0.50	0.15	10.91	<0.001	1.64 (1.22–2.21) *
Shift to telecommuting	0.10	0.09	1.03	0.309	1.10 (0.92–1.32)
Change in food ad exposure	0.42	0.07	35.70	<0.001	1.52 (1.32–1.74) *
Perceived stress scale score	0.04	0.01	12.39	<0.001	1.04 (1.02–1.07) *
Perceived current health	0.42	0.09	24.45	<0.001	1.53 (1.29–1.81) *
Model χ^2^ = 153.14, *p* < 0.001 Hosmer and Lemeshow χ^2^ = 5.93, *p* = 0.655 Pseudo R^2^ = 0.197 *n* = 958					

* indicates statistical significance at the *p* < 0.05 level.

## Data Availability

The data presented in this study are available from the corresponding author upon reasonable request.

## Supplementary Materials

The following are available online at https://www.mdpi.com/article/10.3390/nu13062097/s1, Table S1. Binary logistic regression analysis of perceived increase in dietary healthfulness among males (*n* = 491); Table S2. Binary logistic regression analysis of perceived increase in dietary healthfulness among females (*n* = 467); Table S3. Binary logistic regression analysis of perceived increase in dietary healthfulness among individuals aged 18-29 years (*n* = 205); Table S4. Binary logistic regression analysis of perceived increase in dietary healthfulness among individuals aged 30-39 years (*n* = 331); Table S5. Binary logistic regression analysis of perceived increase in dietary healthfulness among individuals aged 40-49 years (*n* = 200); Table S6. Binary logistic regression analysis of perceived increase in dietary healthfulness among individuals aged 50-59 years (*n* = 120); Table S7. Binary logistic regression analysis of perceived increase in dietary healthfulness among individuals aged 60+ years (*n* = 102); File S1: COVID-19 and Health Behaviors Questionnaire.
