# Supplementary material for: Predictors of COVID-19-Related Perceived Improvements in Dietary Health: Results from a US Cross-Sectional Study
Source: Nutrients. 2021 Jun 19;13(6):2097. doi: 10.3390/nu13062097 (PMC8234783; doi:10.3390/nu13062097)
Supplement: Supplementary file 1 [file nutrients-13-02097-s001.zip › COVID health subgroup analyses.pdf]

Supplementary Table 1. Binary logistic regression analysis of perceived increase in dietary healthfulness among males (n=491).

Independent variable	B	S.E.	Wald	P-value	OR (95% CI)
Household income (2019)	0.06	0.07	0.71	0.399	1.06 (0.93-1.12)
**Change in frequency meals with family in front of the TV**	**0.05**	**0.09**	**0.27**	**0.601**	**1.05 (0.88-1.24)**
COVID-19 income loss	0.48	0.22	4.86	0.028	1.61 (1.05-2.47)*
Shift to telecommuting	-0.14	0.13	1.08	0.298	0.87 (0.67-1.13)
Change in food ad exposure	0.40	0.10	14.80	<0.001	1.49 (1.22-1.83)*
**Perceived Stress Scale score**	**0.03**	**0.02**	**3.84**	**0.05**	**1.03 (1.00-1.07)**
Perceived current health	0.41	0.12	11.33	<0.001	1.51 (1.19-1.91)*

Model χ^2 = 66.602 P <0.001
Hosmer and Lemeshow χ^2 =11.485 P=0.176
Pseudo R^2= 0.169
n= 491

*indicates statistical significance at the p<0.05 level

Statistical conclusions between the regressions in the total study sample and the subgroup (shown above) differ for the variables in bold font

Supplementary Table 2. Binary logistic regression analysis of perceived increase in dietary healthfulness among females (n=467).

Independent variable	B	S.E.	Wald	P-value	OR (95% CI)
Household income (2019)	0.01	0.06	0.01	0.920	1.00 (0.89-1.14)
Change in frequency meals with family in front of the TV	0.30	0.10	9.38	0.002	1.35 (1.11-1.63)*
COVID-19 income loss	0.52	0.21	6.01	0.014	1.68 (1.11-2.55)*
Shift to telecommuting	-0.05	0.13	1.34	0.715	0.95 (0.73-1.24)
Change in food ad exposure	0.44	0.10	20.94	<0.001	1.56 (1.29-1.88)*
Perceived Stress Scale score	0.05	0.02	9.09	0.003	1.05 (1.02-1.08)*
Perceived current health	0.45	0.12	13.37	<0.001	1.56 (1.23-1.98)*

Model χ^2 = 81.986 P <0.001
Hosmer and Lemeshow χ^2 =12.879 P=0.116
Pseudo R^2= 0.216
n= 467

*indicates statistical significance at the p<0.05 level

Statistical conclusions between the regressions in the total study sample and the subgroup (shown above) differ for the variables in bold font

Supplementary Table 3. Binary logistic regression analysis of perceived increase in dietary healthfulness among individuals aged 18-29 years (n=205).

Independent variable	B	S.E.	Wald	P-value	OR (95% CI)
Sex	-0.31	0.32	0.99	0.320	0.73 (0.40-1.36)
Household income (2019)	0.09	0.10	0.82	0.365	1.09 (0.90-1.32)

Change in frequency meals with family in front of the TV	0.39	0.14	8.24	0.004	1.48 (1.13-1.94)*
COVID-19 income loss	0.77	0.33	5.37	0.020	2.16 (1.13-4.13)*
Shift to telecommuting	-0.09	0.21	0.17	0.681	0.92 (0.61-1.39)
**Change in food ad exposure**	**0.14**	**0.17**	**0.64**	**0.424**	**1.15 (0.82-1.61)**
**Perceived Stress Scale score**	**0.04**	**0.03**	**1.53**	**0.216**	**1.04 (0.98-1.11)**
Perceived current health	0.44	0.19	5.30	0.021	1.55 (1.07-2.25)*

Model χ^2 = 32.488 P <0.001
Hosmer and Lemeshow χ^2 =4.767 P=0.782
Pseudo R^2= 0.195
n=205

*indicates statistical significance at the p<0.05 level

Statistical conclusions between the regressions in the total study sample and the subgroup (shown above) differ for the variables in bold font

Supplementary Table 4. Binary logistic regression analysis of perceived increase in dietary healthfulness among individuals aged 30-39 years (n=331).

Independent variable	B	S.E.	Wald	P-value	OR (95% CI)
Sex	0.06	0.24	0.07	0.793	1.07 (0.66-1.71)
Household income (2019)	-0.03	0.08	0.13	0.718	0.97 (0.84-1.13)
**Change in frequency meals with family in front of the TV**	**0.08**	**0.10**	**0.70**	**0.402**	**1.09 (0.89-1.32)**
**COVID-19 income loss**	**0.24**	**0.25**	**0.94**	**0.332**	**1.27 (0.78-2.07)**
Shift to telecommuting	-0.09	0.17	0.27	0.603	0.92 (0.66-1.27)
Change in food ad exposure	0.32	0.12	7.68	0.006	1.38 (1.10-1.72)*
**Perceived Stress Scale score**	**0.04**	**0.02**	**3.23**	**0.072**	**1.04 (1.00-1.08)**
Perceived current health	0.51	0.15	11.40	<0.001	1.66 (1.24-2.22)*

Model χ^2 = 36.011 P <0.001
Hosmer and Lemeshow χ^2 =5.046 P=0.753
Pseudo R^2= 0.138
n=331

*indicates statistical significance at the p<0.05 level

Statistical conclusions between the regressions in the total study sample and the subgroup (shown above) differ for the variables in bold font

Supplementary Table 5. Binary logistic regression analysis of perceived increase in dietary healthfulness among individuals aged 40-49 years (n=200).

Independent variable	B	S.E.	Wald	P-value	OR (95% CI)
Sex	-0.12	0.33	0.13	0.714	0.89 (0.47-1.68)
Household income (2019)	0.02	0.10	0.03	0.856	1.02 (0.84-1.24)
**Change in frequency meals with family in front of the TV**	**0.13**	**0.16**	**0.65**	**0.422**	**1.13 (0.84-1.54)**
**COVID-19 income loss**	**0.34**	**0.34**	**0.96**	**0.327**	**1.40 (0.71-2.75)**

Shift to telecommuting	0.01	0.20	0.01	0.944	1.01 (0.68-1.51)
Change in food ad exposure	0.70	0.16	18.92	<0.001	2.00 (1.47-2.76)*
Perceived Stress Scale score	0.05	0.03	3.86	0.049	1.05 (1.00-1.11)*
Perceived current health	0.53	0.19	7.58	0.006	1.70 (1.17-2.48)*
Model χ^2 = 46.165 P <0.001					
Hosmer and Lemeshow χ^2 =15.105 P=0.057					
Pseudo R^2= 0.275					
n=200					

*indicates statistical significance at the p<0.05 level

Statistical conclusions between the regressions in the total study sample and the subgroup (shown above) differ for the variables in bold font

Supplementary Table 6. Binary logistic regression analysis of perceived increase in dietary healthfulness among individuals aged 50-59 years (n=120).

Independent variable	B	S.E.	Wald	P-value	OR (95% CI)
Sex	-0.74	0.45	2.69	0.101	0.48 (0.20-1.15)
Household income (2019)	0.12	0.12	0.94	0.332	1.13 (0.89-1.43)
**Change in frequency meals with family in front of the TV**	**0.23**	**0.23**	**1.02**	**0.313**	**1.26 (0.80-2.00)**
**COVID-19 income loss**	**0.84**	**0.47**	**3.27**	**0.070**	**2.33 (0.93-5.80)**
Shift to telecommuting	-0.07	0.29	0.05	0.822	0.94 (0.53-1.66)
Change in food ad exposure	0.54	0.19	7.77	0.005	1.72 (1.17-2.51)*
**Perceived Stress Scale score**	**0.06**	**0.04**	**3.01**	**0.083**	**1.06 (0.99-1.14)**
**Perceived current health**	**0.40**	**0.28**	**2.15**	**0.142**	**1.50 (0.87-2.57)**
Model χ^2 = 36.283 P <0.001					
Hosmer and Lemeshow χ^2 =7.728 P=0.460					
Pseudo R^2= 0.349					
n=120					

*indicates statistical significance at the p<0.05 level

Statistical conclusions between the regressions in the total study sample and the subgroup (shown above) differ for the variables in bold font

Supplementary Table 7. Binary logistic regression analysis of perceived increase in dietary healthfulness among individuals aged 60+ years (n=102).

Independent variable	B	S.E.	Wald	P-value	OR (95% CI)
Sex	-0.18	0.50	0.14	0.713	0.83 (0.31-2.21)
Household income (2019)	-0.21	0.17	1.59	0.208	0.81 (0.58-1.13)
**Change in frequency meals with family in front of the TV**	**-0.09**	**0.24**	**0.14**	**0.705**	**0.91 (0.57-1.47)**

COVID-19 income loss	1.28	0.54	5.68	0.017	3.60 (1.26-10.30)*
Shift to telecommuting	-0.41	0.31	1.80	0.180	0.66 (0.36-1.21)
Change in food ad exposure	0.58	0.25	5.30	0.021	1.78 (1.09-2.90)*
**Perceived Stress Scale score**	**0.00**	**0.04**	**0.01**	**0.938**	**1.00 (0.93-1.08)**
**Perceived current health**	**-0.04**	**0.27**	**0.02**	**0.876**	**0.96 (0.57-1.63)**
Model χ^2 = 22.493 P =0.004					
Hosmer and Lemeshow χ^2 =6.509 P=0.590					
Pseudo R^2= 0.275					
n=102					

*indicates statistical significance at the $p<0.05$ level

Statistical conclusions between the regressions in the total study sample and the subgroup (shown above) differ for the variables in bold font
