# Supplementary material for: Predictors of COVID-19-Related Perceived Improvements in Dietary Health: Results from a US Cross-Sectional Study
Source: Nutrients. 2021 Jun 19;13(6):2097. doi: 10.3390/nu13062097 (PMC8234783; doi:10.3390/nu13062097)
Supplement: Supplementary file 1 [file nutrients-13-02097-s001.zip › COVID-19_Household_food_behaviors.pdf]

COVID-19 Household food behaviors

Do you speak/read/write the English language?

- ◯ Yes
- ◯ No

Are you 18 years of age or older?

- ◯ Yes
- ◯ No

Do you currently reside in the United States?

- ◯ Yes
- ◯ No

Do you consider yourself the primary food purchaser in your home (the individual who does most of the grocery shopping)?

- ◯ Yes
- ◯ No

Display This Question:

If Do you speak/read/write the English language? = No

Or Are you 18 years of age or older? = No

Or Do you currently reside in the United States? = No

Or Do you consider yourself the primary food purchaser in your home (the individual who does most of... = No

Unfortunately, you are not eligible to participate in our study. No completion code will be provided.

Display This Question:

If Do you speak/read/write the English language? = Yes

And Are you 18 years of age or older? = Yes

And Do you currently reside in the United States? = Yes

And Do you consider yourself the primary food purchaser in your home (the individual who does most of... = Yes

You are eligible to participate in our study!

Welcome to the COVID-19 and Household Health Behaviors survey. Please take your time to read the questions and choose the answer that best describes your household.

Please start by telling us a little bit about yourself and the people in your household.

How old are you? Please enter a number.

____

What is your sex?

○ Male

○ Female

Are you of Hispanic, Latino, or Spanish origin?

- ○ Yes
- ○ No

What is your race? Please select all that apply.

- ☐ White
- ☐ Black or African American
- ☐ American Indian or Alaska Native
- ☐ Asian
- ☐ Native Hawaiian or Pacific Islander
- ☐ Other

What is the highest degree or level of school you have completed?

- Less than high school
- Some high school
- High school graduate or equivalent
- Some college but degree not received or is in progress
- Associate's degree
- Bachelor's degree
- Graduate degree

What is your marital status?

- Now married
- Widowed
- Divorced
- Separated
- Never married

How many total people – adults and children – currently live in your household, including yourself? Please enter a number.

____

How many people under 18 years old currently live in your household? Please enter a number.

____

In 2019 what was your **total household income** before taxes?

- ○ Less than $25,000
- ○ $25,000 - $34,999
- ○ $35,000 - $49,999
- ○ $50,000 - $74,999
- ○ $75,000 - $99,999
- ○ $100,000 - $149,999
- ○ $150,000 - $199,999
- ○ $200,000 and above

Have you or anyone in your household experienced a loss of employment income due to the COVID-19 pandemic?

- ○ Yes
- ○ No

Are all of the adults in your household employed?

- ◯ Yes
- ◯ No

Display This Question:

If Are all of the adults in your household employed? = No

What are the main reasons for the adult(s) in your household being unemployed?

- ☐ Do not want to be employed at this time
- ☐ Sick with coronavirus symptoms
- ☐ Caring for someone with coronavirus symptoms
- ☐ Caring for children not in school or daycare
- ☐ Caring for an elderly person
- ☐ Concerned about getting or spreading the coronavirus
- ☐ Sick (not coronavirus related) or disabled
- ☐ Retired
- ☐ Employer experienced a reduction in business (including furlough) due to coronavirus pandemic
- ☐ Laid off due to coronavirus pandemic
- ☐ Employer closed temporarily due to the coronavirus pandemic
- ☐ Employer went out of business due to the coronavirus pandemic
- ☐ Other reason, please specify

____

Working from home is sometimes referred to as telework. Did any adults in your household, including yourself, substitute some or all of their typical in-person work for telework because of the COVID-19 pandemic?

- ○ Yes, at least one adult substituted **all** of their typical in-person work for telework
- ○ Yes, at least one adult substituted **some** of their typical in-person work for telework
- ○ No, no adults substituted their typical in-person work for telework

Display This Question:

If If How many people under 18 years old currently live in your household? Please enter a number. Text Response Is Greater Than 0

Are any adults in your household responsible for caring for children who are staying at home **because of the COVID-19 pandemic**?

- ○ Yes
- ○ No

Now we are going to ask about the food situation in your household.

Please indicate whether the following statements were true **before the COVID-19 pandemic**.

	Declined to answer	Never true	Sometimes true	Often true
I worried whether our food would run out before I got money to buy more	○	○	○	○
The food that I bought just didn't last, and I didn't have money to get more	○	○	○	○
I couldn't afford to eat balanced meals	○	○	○	○

Please indicate whether the following statements have been true **during the COVID-19 pandemic**.

	Declined to answer	Never true	Sometimes true	Often true
I worried whether our food would run out before I got money to buy more	◯	◯	◯	◯
The food that I bought just didn't last, and I didn't have money to get more	◯	◯	◯	◯
I couldn't afford to eat balanced meals	◯	◯	◯	◯

Because of the COVID-19 pandemic, did you stock up on nonperishable food?
NOTE: nonperishable food includes shelf-stable foods that do not require refrigeration

◯ Yes

◯ No

Display This Question:

If Because of the COVID-19 pandemic, did you stock up on nonperishable food? NOTE: nonperishable foo... = Yes

When you stocked up on nonperishable food, how much extra food did you purchase compared to the amount you normally buy?

- Much more
- Moderately more
- Slightly more

Display This Question:

If Because of the COVID-19 pandemic, did you stock up on nonperishable food? NOTE: nonperishable foo... = Yes

Of the nonperishable food you purchased when stocking up, how much **has been** consumed by someone in your household?

- All
- Most
- Some
- Very little
- None

Display This Question:

If Because of the COVID-19 pandemic, did you stock up on nonperishable food? NOTE: nonperishable foo... = Yes

Of the nonperishable food you purchased when stocking up, how much do you think **will eventually be** consumed by someone in your household?

- ○ All
- ○ Most
- ○ Some
- ○ Very little
- ○ None

Before the COVID-19 pandemic, where did you obtain most of the food for household consumption? Check all that apply.

- ☐ Grocery store
- ☐ Farmer's market
- ☐ Convenience store
- ☐ Online grocery shopping
- ☐ Takeout (pick up or home delivery)
- ☐ Sit-down restaurant
- ☐ Fast food restaurant
- ☐ Meal kit service (Blue Apron, Hello Fresh, etc.)
- ☐ Home garden
- ☐ Other ______________________________

During the COVID-19 pandemic, where have you obtained most of the food for household consumption? Check all that apply.

- [ ] Grocery store
- [ ] Farmer's market
- [ ] Convenience store
- [ ] Online grocery shopping
- [ ] Takeout (pick up or home delivery)
- [ ] Sit-down restaurant
- [ ] Fast food restaurant
- [ ] Meal kit service (Blue Apron, Hello Fresh, etc.)
- [ ] Home garden
- [ ] Other ____

Before the COVID-19 pandemic, how often did you or someone in your household eat something from a fast food restaurant (McDonald's, Burger King, etc.) in a typical week?

- ◯ Never
- ◯ 1-2 times
- ◯ 3-4 times
- ◯ 5-6 times
- ◯ 7 times
- ◯ More than 7 times

During the COVID-19 pandemic, how often have you or someone in your household eaten something from a fast food restaurant (McDonald's, Burger King, etc.) in a typical week?

- ◯ Never
- ◯ 1-2 times
- ◯ 3-4 times
- ◯ 5-6 times
- ◯ 7 times
- ◯ More than 7 times

Before the COVID-19 pandemic, what percentage of meals consumed in your household were prepared at home?

	0 10 20 30 40 50 60 70 80 90 100
Percentage of meals prepared at home	

During the COVID-19 pandemic, what percentage of meals consumed in your household have been prepared at home?

	0 10 20 30 40 50 60 70 80 90 100
Percentage of meals prepared at home	

Compared to your habits prior to the COVID-19 pandemic, how have your grocery shopping behaviors changed during the pandemic?

	Buy significantly less	Buy somewhat less	No change	Buy somewhat more	Buy significantly more
Fresh fruit					
Fresh vegetables					
Dairy products					
Meat/poultry					
Eggs					
Meat alternatives (veggie burgers, tofu, etc.)					
Seafood					
Nuts and seeds					
Grains/flour and legumes					
Bread/bread products					
Nonperishable foods					
Frozen foods					

<u>Compared to your habits prior to the COVID-19 pandemic</u>, how have your food waste behaviors changed during the pandemic?

	Waste significantly less	Waste somewhat less	No change	Waste somewhat more	Waste significantly more
Fresh fruit	○	○	○	○	○
Fresh vegetables	○	○	○	○	○
Dairy products	○	○	○	○	○
Meat/poultry	○	○	○	○	○
Eggs	○	○	○	○	○
Meat alternatives (veggie burgers, tofu, etc.)	○	○	○	○	○
Seafood	○	○	○	○	○
Nuts and seeds	○	○	○	○	○
Grains/flour and legumes	○	○	○	○	○
Bread/bread products	○	○	○	○	○
Nonperishable foods	○	○	○	○	○
Frozen foods	○	○	○	○	○

Before the COVID-19 pandemic, in an average week, what percentage of food purchased at the **grocery store** would you usually throw away?

	0 10 20 30 40 50 60 70 80 90 100
Percentage of groceries thrown away	

During the COVID-19 pandemic, in an average week, what percentage of food purchased at the **grocery store** do you usually throw away?

	0 10 20 30 40 50 60 70 80 90 100
Percentage of groceries thrown away	

Before the COVID-19 pandemic, in an average week, what percentage of food purchased from a **restaurant** (including takeout) would usually get thrown away (for example, leftovers that don't get eaten or food that gets left on the plate at the restaurant)?

	0 10 20 30 40 50 60 70 80 90 100
Percentage of food wasted	

During the COVID-19 pandemic, in an average week, what percentage of food purchased from a **restaurant** (including takeout) usually gets thrown away (for example, leftovers that don't get eaten or food that gets left on the plate at the restaurant)?

	0 10 20 30 40 50 60 70 80 90 100
Percentage of food wasted	

You are almost done with all questions. By clicking "Somewhat disagree" you confirm that you are still paying attention to this survey.

- Strongly agree
- Agree
- Somewhat agree
- Neither agree nor disagree
- Somewhat disagree
- Disagree
- Strongly disagree

Display This Question:

If You are almost done with all questions. By clicking "Somewhat disagree" you confirm that you are... != Somewhat disagree

Unfortunately, you did not pass our check question. No completion code will be provided.

Skip To: End of Survey If Unfortunately, you did not pass our check question. No completion code will be provided. Is Displayed

During the COVID-19 pandemic, have you or anyone in your household been exposed to more food advertisements than before the pandemic?

- ○ Definitely yes
- ○ Probably yes
- ○ Might or might not
- ○ Probably not
- ○ Definitely not

Did the eating habits of anyone in your household change because of the COVID-19 pandemic?

- ○ Definitely yes
- ○ Probably yes
- ○ Might or might not
- ○ Probably not
- ○ Definitely not

Display This Question:

If Did the eating habits of anyone in your household change because of the COVID-19 pandemic? != Definitely not

And Did the eating habits of anyone in your household change because of the COVID-19 pandemic? != Probably not

How would you describe the impact of those changes on the healthfulness of eating habits in your household?

- ○ Eating habits became much healthier
- ○ Eating habits became somewhat healthier
- ○ Eating habits became neither healthier nor less healthy
- ○ Eating habits became somewhat less healthy
- ○ Eating habits became much less healthy

Display This Question:

If Did the eating habits of anyone in your household change because of the COVID-19 pandemic? != Definitely not

And Did the eating habits of anyone in your household change because of the COVID-19 pandemic? != Probably not

Why did eating habits change in your household? Check all that apply.

- ☐ Financial limitations
- ☐ More time to cook
- ☐ Less time to cook
- ☐ Less access to restaurants
- ☐ Increased stress/emotional eating
- ☐ Other ____

How do you perceive your current health?

- ○ Excellent
- ○ Very good
- ○ Good
- ○ Fair
- ○ Poor

Please answer the following questions.

	Never	Almost never	Sometimes	Fairly often	Often
In the last month, how often have you been upset because of something that happened unexpectedly?	○	○	○	○	○
In the last month, how often have you felt that you were unable to control the important things in your life?	○	○	○	○	○
In the last month, how often have you felt nervous and stressed?	○	○	○	○	○
In the last month, how often have you felt confident about your ability to handle your personal problems?	○	○	○	○	○
In the last month, how often have you felt that things were going your way?	○	○	○	○	○
In the last month, how	○	○	○	○	○

often have you found that you could not cope with all the things that you had to do?					
In the last month, how often have you been able to control irritations in your life?	○	○	○	○	○
In the last month, how often have you felt that you were on top of things?	○	○	○	○	○
In the last month, how often have you been angered because of things that happened that were outside of your control?	○	○	○	○	○
In the last month, how often have you felt difficulties were piling up so high that you could not overcome them?	○	○	○	○	○

Almost done. Now please tell us a little bit about your screen-time habits.

Before the COVID-19 pandemic, how many times in a typical week did all, or most, of the people living in your house eat a meal together **without** any screens on?

- Never
- 1-2 times
- 3-4 times
- 5-6 times
- 7 times
- More than 7 times

During the COVID-19 pandemic, how many times in a typical week do all, or most, of the people living in your house eat a meal together **without** any screens on?

- Never
- 1-2 times
- 3-4 times
- 5-6 times
- 7 times
- More than 7 times

Before the COVID-19 pandemic, how many times in a typical week did all, or most, of the people living in your house eat a meal together **while watching television**?

- Never
- 1-2 times
- 3-4 times
- 5-6 times
- 7 times
- More than 7 times

During the COVID-19 pandemic, how many times in a typical week do all, or most, of the people living in your house eat a meal together **while watching television**?

- Never
- 1-2 times
- 3-4 times
- 5-6 times
- 7 times
- More than 7 times

Thank you for your participation. If you are interested in participating in health-related surveys, look for more HITS from our research group in the near future.
